# Decoding the language of first impressions: Comparing models of first impressions of faces derived from free‐text descriptions and trait ratings

**DOI:** 10.1111/bjop.12717

**Published:** 2024-06-17

**Authors:** Alex L. Jones, Victor Shiramizu, Benedict C. Jones

**Affiliations:** ^1^ School of Psychology Swansea University Swansea UK; ^2^ Department of Psychological Sciences & Health University of Strathclyde Glasgow Scotland

**Keywords:** computational modelling, methodology, person perception, perception, social cognition

## Abstract

First impressions formed from facial appearance predict important social outcomes. Existing models of these impressions indicate they are underpinned by dimensions of Valence and Dominance, and are typically derived by applying data reduction methods to explicit ratings of faces for a range of traits. However, this approach is potentially problematic because the trait ratings may not fully capture the dimensions on which people spontaneously assess faces. Here, we used natural language processing to extract ‘topics’ directly from participants' free‐text descriptions (i.e., their first impressions) of 2222 face images. Two topics emerged, reflecting first impressions related to positive emotional valence and warmth (Topic 1) and negative emotional valence and potential threat (Topic 2). Next, we investigated how these topics were related to Valence and Dominance components derived from explicit trait ratings. Collectively, these components explained only ~44% of the variance in the topics extracted from free‐text descriptions and suggested that first impressions are underpinned by correlated valence dimensions that subsume the content of existing trait‐rating‐based models. Natural language offers a promising new avenue for understanding social cognition, and future work can examine the predictive utility of natural language and traditional data‐driven models for impressions in varying social contexts.

## BACKGROUND

First impressions based on facial appearance play an important role in social interaction (Todorov et al., 2015; Zebrowitz & Montepare, 2008). Impressions of faces on a wide range of traits (e.g., trustworthiness, competence and attractiveness) influence real‐world social outcomes (Langlois et al., 2000; Rhodes, 2006; Todorov et al., 2015) and are formed rapidly and automatically (Borkenau et al., 2009; Eggleston et al., 2021; Olivola & Todorov, 2010; Willis & Todorov, 2006). For example, first impressions based on facial appearance predict election outcomes (Ballew & Todorov, 2007; Olivola & Todorov, 2010; Olivola et al., 2012), judicial decisions (Dumas & Testé, 2006; Olivola, Funk, & Todorov, 2014; Wilson & Rule, 2015), partner choice (South Palomares & Young, 2018), hiring decisions (Olivola, Eubanks, & Lovelace, 2014; Zebrowitz & Montepare, 2008) and many aspects of economic exchange (Gheorghiu et al., 2017; Jaeger et al., 2019; Menegatti et al., 2021; van't Wout & Sanfey, 2008). Thus, understanding the processes and mechanisms that underlie first impressions of faces can provide insight into an important driver of social interactions and outcomes (Olivola, Funk, & Todorov, 2014; Todorov et al., 2015; Zebrowitz & Montepare, 2008).

Research on first impressions formed from faces has investigated a diverse range of traits, such as trustworthiness (Jaeger et al., 2018; Stirrat & Perrett, 2010; Todorov, 2008; Todorov & Duchaine, 2008), dominance (Fruhen et al., 2015; Hester et al., 2021; Quist et al., 2011), attractiveness (Holzleitner et al., 2019; Jones & Jaeger, 2019), competence (Oh et al., 2019; Todorov et al., 2005), aggressiveness (Lefevre & Lewis, 2014), intelligence (Kleisner et al., 2014; Talamas et al., 2016) and sociability (Jaeger et al., 2022; Mehu et al., 2007). However, arguably the most important advances in the first‐impressions literature have come from studies that used data‐reduction methods to identify the core perceptual dimensions that underpin the wide range of often intercorrelated traits that are typically considered in the literature (Todorov et al., 2013). Moreover, Oosterhof and Todorov's (2008) ‘valence–dominance’ model, which was derived using this approach, has been particularly influential in the first‐impressions literature.

The initial description of the valence–dominance model (Oosterhof & Todorov, 2008) first asked 55 participants to provide spontaneous descriptions for each of 66 faces. The researchers then sorted the 1134 descriptions produced at this initial step into 14 trait categories and, importantly for the current work, removed any descriptors that were unrelated to personality traits. They then assigned each of these categories a trait label, and had the 66 faces rated for each of these labels (plus the additional trait ‘dominance’) by a different group of participants. Principal Component Analysis (PCA) of these explicit trait ratings revealed two components that explained 81.6% of the variance in the explicit trait ratings. The first component, labelled Valence, was highly correlated with ratings of traits such as trustworthiness, emotional stability, responsibility, caringness and sociability, and was interpreted as reflecting impressions of other people's prosocial intentions. The second component, labelled Dominance, was highly correlated with ratings of traits such as dominance, aggressiveness and meanness, and was interpreted as reflecting impressions of other peoples' capacity to inflict harm on the perceiver. Although studies of explicit ratings of faces, bodies and voices in which stimuli were rated for the same (or conceptually similar) traits to those considered by Oosterhof and Todorov (2008) have also concluded that first impressions are underpinned by Valence and Dominance dimensions (Jones & Kramer, 2021; McAleer et al., 2014; Morrison et al., 2017; Shiramizu et al., 2022; Tzschaschel et al., 2022), some other studies that investigated different traits and/or used different data‐reduction methods have observed additional dimensions (e.g., a youthfulness dimension) that also contributed to first impressions (Lin et al., 2021; Sutherland et al., 2013). Evidence for the valence–dominance model has also come from cross‐cultural research, at least when the analytical methods employed were the same as those used by Oosterhof and Todorov (Jones et al., 2021).

A potentially important limitation of the studies described above is that, in each case, the dimensions underpinning first impressions were identified by analysing explicit trait ratings of stimuli (i.e., participants were explicitly instructed to rate stimuli for traits that were specified by the researchers). However, there is growing concern that such traits may not necessarily fully capture the richness of first impressions, as researchers decide what traits participants will rate the faces for (Mondloch et al., 2023; Satchell et al., 2022; Sutherland & Young, 2022). Indeed, some researchers have argued that it may be more useful to analyse types of responses or data that are less constrained, and potentially influenced, by researcher expectations and assumptions (Jack et al., 2018; Satchell et al., 2022).

In light of the above, we first used natural language processing techniques (NLP) to analyse participants' free‐text descriptions of 2222 face images (Study 1). These techniques derive statistical regularities from text data, extracting patterns of words or phrases that occur in documents (Ding et al., 2023; Liu et al., 2021) that represent the core ‘topics’ that might underpin participants' written descriptions (i.e., first impressions) of the individuals shown in the images. In Study 2, we investigated the extent to which the extracted topics could be predicted from valence and dominance components derived from the same explicit trait ratings considered in previous studies of the valence–dominance model. We carried out Study 2 to quantify the similarity between the topics extracted from free‐text descriptions of faces in Study 1 and the valence and dominance components derived from explicit trait ratings.

## STUDY ONE – TOPIC MODELLING OF FIRST IMPRESSIONS

To identify the core dimensions (or topics) underpinning free text descriptions (i.e., first impressions) of faces, we employed topic modelling with a large, open‐access face image set (the 10k US Adult Faces Database, Bainbridge et al., 2013). This approach allows us to use topic modelling in an analogous way to the dimension reduction techniques applied to explicit trait rating data in previous work on the dimensions underpinning first impressions of faces.

### Method

#### Stimuli

The 10k US Adult Faces Database (Bainbridge et al., 2013) contains naturalistic images of 2222 adult face images, captured in unconstrained natural poses. Faces vary in ethnicity (83.7% White, 9.9% Black, 3.2% Hispanic and 3.1% Asian), sex (42.9% female), age, emotional expression and pose.

#### Procedure and participants

To collect free‐text descriptions (i.e., written first impressions) of the images while reducing the potential for participant fatigue, 22 sets of faces (each set consisting of 101 images) were first created by random sampling from the face‐image database. Two hundred and forty‐four participants (140 women and 100 men, mean age = 40.10 years, *SD* = 13.14 years) were then randomly allocated to one of these image sets. In each image set, participants were sequentially presented with a face and a text box and were asked to describe their first impressions of that person by entering text into the text box. Participants were also instructed that they could use whatever words came to mind and however many words they wanted. The trial order (i.e., the order in which faces were presented) was fully randomized and the study was self‐paced. The study was run online and participants were recruited via Prolific (constrained to the United Kingdom and United States, with English as a first language), and data were collected using the Gorilla online testing platform (Anwyl‐Irvine et al., 2020). Each face received free‐text descriptions from an average of 10.50 participants (*SD* = 0.89).

#### Text processing pipeline

Topic modelling is an unsupervised learning approach that is capable of parsing sets of documents, or a corpus, to automatically cluster together words or phrases that have high semantic coherence (Steyvers & Griffiths, 2014). Moving from raw text to topic extraction requires pre‐processing steps, highlighted here.

First, a single document file for each face was produced by concatenating all descriptions given for that face into a single document or paragraph. Text such as digits and punctuation (for example, exclamation or question marks) were removed, clear misspellings of words were corrected and all text was converted to lowercase. Stop‐words were then removed (e.g., function words like ‘always’, ‘because’ and ‘but’). Each document was then lemmatized – that is, words are replaced with their *lemma*, or the most basic representation of a set of word forms – for example, the words ‘feels’, ‘felt’ and ‘feeling’ have the lemma ‘*feel*’. Lemmatized words are words removed from cues that help form sentence structure, or offer information about temporal context (e.g. *felt* indicating past tense). Practically, lemmatization reduces variation in text data and makes it easier to capture the meaning of words present in a text. Instead of counting instances of words such as *‘smiling’* and *‘smiled’* as separate words, the count of the lemma *‘smile*’ is obtained.

The processed documents were converted from text to a matrix representation using a two‐step process. The entire set of lemmatized documents (or corpus) was initially converted into a bag‐of‐words (BOW) representation. All individual words across every document (here, a description for a face) are isolated, indicating the number of unique words in the corpus. The number of times each word appears within each document (face description) is counted. This creates a sparse matrix representation with documents as rows, columns as words and entries as non‐negative counts, capturing the occurrence of words in documents. While this representation discards word order, it has the distinct benefits of simple computation and being directly interpretable – each column is tied to the instance of a word. The BOW representation was then rescaled using the term‐frequency inverse‐document‐frequency (TF‐IDF) method. TF‐IDF scales the count representation of each word according to how often it appears in each document relative to its appearance in other documents. Higher values in the matrix represent a word that is used more frequently in that document, but not others.

To extract topics, the TF‐IDF representation of the corpus was subjected to Non‐Negative Matrix Factorization (NMF; Lee & Seung, 1999). NMF takes an observed *n* x *p* matrix (*n* documents, *p* words), a predefined number of components, and estimates two matrices (*H* and *W*), which, when multiplied, return the observed matrix (with some error). The *H* and *W* matrices have useful properties for topic modelling, as they naturally cluster together words into topics and assign document affinities for a given topic. For example, for the extraction of three components, NMF would yield *H*, a *p* x 3 matrix where higher entries would indicate greater association between words and the component, and *W*, an *n* x 3 matrix where higher entries indicate greater association between a document and the components. The components can be given meaning (i.e. topics) by examining the words with the largest entries in *H* and the representative documents with the highest entries in *W*.

NMF requires an a priori number of components to extract. Given that this number of components/topics ties directly to the conclusions about the psychological structure of first impressions, we sought to evaluate the quality of the NMF matrix estimations using an objective topic coherence measure from computational linguistics, the UMass score (Mimno et al., 2011). The UMass score computes the log probability of observing a given pair of words in a random document in the corpus, divided by the number of documents, computed across all possible pairings of words in a topic. Scores closer to zero are more coherent (Mimno et al., 2011; Rüdiger et al., 2022). Based on the number of components found in previous ratings‐based studies, we evaluated topic coherence in NMF solutions with two to six components. We limited the coherence score to consider the top 20 words of the resulting components, a reasonable default that topic modelling algorithms show stable correlations with human‐derived topics from text corpora (Arun et al., 2010; Řehůřek & Sojka, 2010; Röder et al., 2015). The average UMass coherence across components was used to select the final number of components/topics.

The code and data underpinning these analyses are available at the OSF (osf.io/chxnp). Text processing and NMF were carried out with Python and the scikit‐learn package (Pedregosa et al., 2011).

### Results

#### Corpus characteristics

Participants, on average, tended to provide relatively short raw text descriptions (mean number of words per description = 2.55 words, *SD* = 2.44, median = 2). After combining responses for a description of each face, each face had descriptions of on average 26.79 words in length (*SD* = 8.68, median = 25). Importantly, NMF has previously been shown to work well for topic modelling with even short text samples (Albalawi et al., 2020). Moreover, a TF‐IDF representation of text has also shown good results for topic modelling on short document corpuses, such as social media posts (Lossio‐Ventura et al., 2019).

**FIGURE 1:**
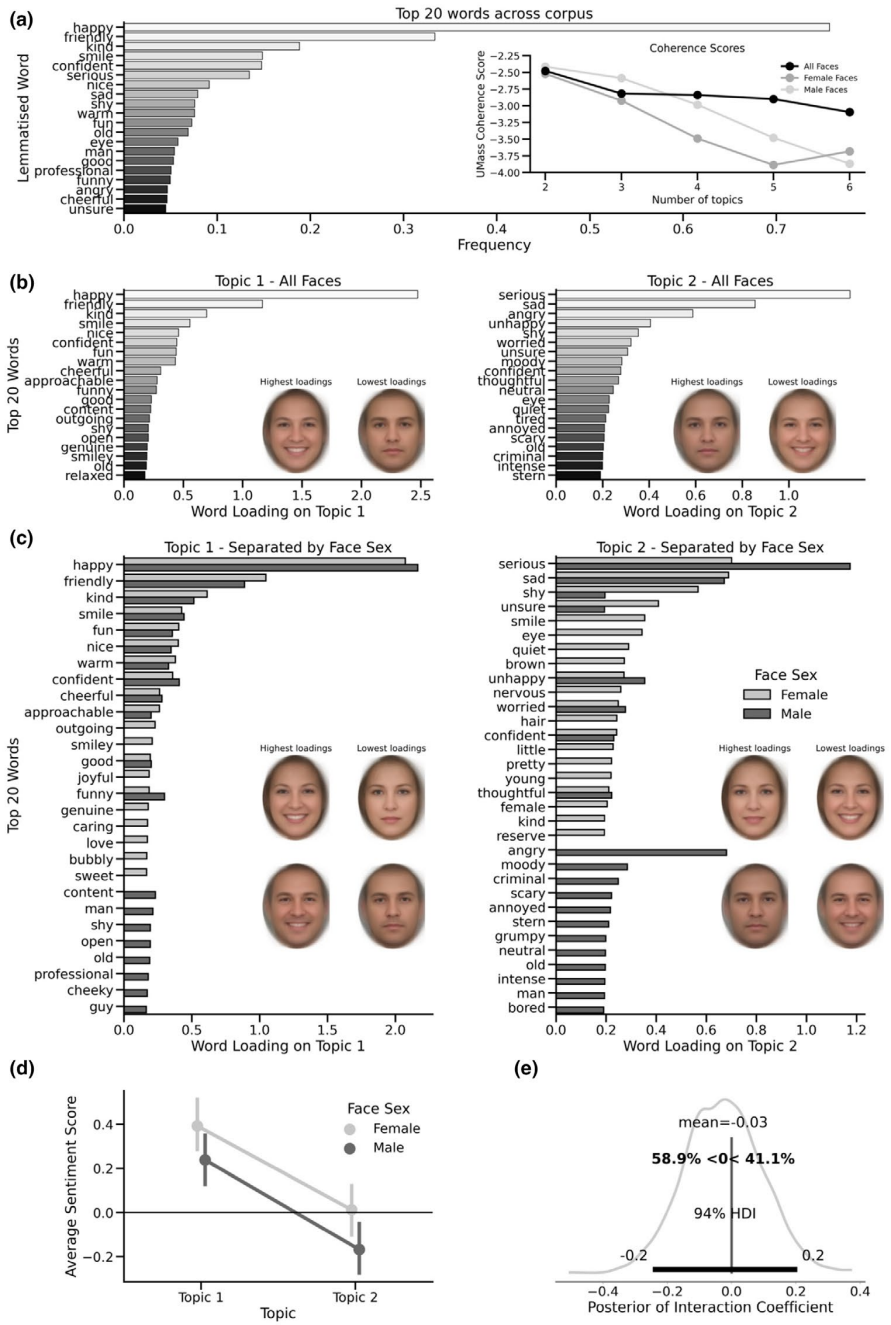
Panel (a) shows the 20 most frequent words across the entire corpus. The inset shows coherence scores for candidate topic numbers for the entire dataset and topics within each sex. Panel (b) shows word loadings for each of the two topics extracted from the entire dataset, including composite faces. Panel (c) shows word loadings for two topics extracted from female and male faces separately and the associated facial appearances. Panel (d) shows average sentiment scores and posterior credible intervals within each topic, as derived from each sex. Panel (e) shows the posterior distribution of the interaction coefficient and the probability that males have lower (more negative) sentiment under Topic 2 as compared to females.

There were a total of 36,006 words extracted across these descriptions, which comprised 4612 unique word entries. Figure 1a illustrates the frequencies of the top 20 words, which shows a concentration of word usage around positive words such as *happy, friendly* and *kind*. This matrix was then subjected to NMF for topic extraction.

#### Topic modelling

NMF was repeated for two to six topics, and at each iteration, the UMass coherence score was calculated on the 20 words with the highest loading on each topic to establish the most coherent set of word‐to‐topic loadings. These scores indicated that a model with two topics produced the most coherent fit (two topics UMass = −2.48, three topics UMass = −2.82, four topics UMass = −2.84, five topics UMass = −2.90, six topics UMass = −3.10; see Figure 1a). Split‐half cross‐validation of this showed strong support for this two‐topic solution (see Supplementary Materials), showing cross‐validated Ochiai coefficients for Topics 1 and 2 of .85 and .66, respectively.

Figure 1b shows the loadings for the 20 words, with the highest loadings for each topic. The words *‘happy’, ‘friendly’, ‘kind’, ‘smile’* and ‘*nice’* showed the highest loadings on Topic 1, suggesting Topic 1 reflected first impressions related to aspects of positive emotional valence, warmth and approachability. By contrast, the words *‘serious’, ‘sad’, ‘angry’, ‘unhappy’* and *‘shy’* showed the highest loadings on Topic 2, suggesting Topic 2 reflected first impressions related to negative emotional valence and potential threat (Topic 2). Using the loading each face received on each topic, we carried out a Bayesian correlation (with a flat prior on the correlation coefficient), to estimate the degree of association between each topic, *r* = −.65, 94% Credible Interval [−0.67, −0.62], indicating that a higher loading on Topic 1 suggests a generally lower loading on Topic 2. We created average faces based on the faces with the highest (*n* = 100) and lowest (*n* = 100) loadings on each topic. These visualizations are shown in Figure 1b and illustrate how cues of positive facial affect (e.g. smiling) are associated with Topic 1 and how more neutral and masculine cues are associated with Topic 2.

We also replicated this analysis using a different topic modelling method, Latent Semantic Analysis (LSA), which uses singular‐value decomposition to extract topics. This alternative approach has a different structure that forces orthogonal components as well as allowing for positive and negative loadings. This approach also confirmed a two‐topic structure, and the resulting topics showed minimal differences to those extracted by NMF (see Supplementary Materials).

#### Topic modelling conditional on face sex

The two‐topic structure described above was generated from both female and male faces. An important possibility to consider is whether a fundamentally different topic structure might emerge when considering female and male faces in isolation. To test this, we repeated the entire analysis above, building a separate corpus for male (*n* = 1267) and female (*n* = 955) faces separately and subjecting those to topic modelling. For female faces, a total of 15,309 words were used, with 2640 unique entries. For males, 20,697 words were used in total, with 3457 unique entries.

For both the female and male faces corpora, a two‐topic solution emerged consistently (females; two topics UMass = −2.52, three topics UMass = −2.92, males; two topics UMass = −2.41, three topics UMass = −2.59; see Figure 1a). As with the full dataset, the topics derived from analysing descriptions of female and male faces separately showed a negative correlation (female faces: *r* = −0.64 [−0.67, −0.60]; male faces: *r* = −0.65 [−0.68, −0.62]). Topics for male and female faces showed broad similarities, albeit with some clear differences. Figure 1c illustrates the top 20 words loading on each topic for both female and male topic models. For Topic 1, there is broad similarity between the highest‐loaded words – for example, the words *happy, friendly, kind, smile* and *fun* had the highest loading for female faces, and *happy, friendly, kind, smile* and *confident* were the highest for males, while only female faces had loadings for words like *love, sweet, caring* and *bubbly*. Conversely, male faces only had positive loadings for words like *professional, content, open* and *shy*. Thus, while Topic 1 seems to broadly represent the same kind of positively‐valanced impressions for both sexes, there are some differences between sexes in the language used.

Topic 2 broadly indexed negatively valanced, threat‐related impressions. For female faces, the words *serious, sad, eye, shy* and *unsure* had the highest loadings, while for male faces, the words *serious, angry, sad, unhappy* and *moody* had the highest loadings. Notably, there were more unique words associated with each topic for each sex. Female faces had loadings for words like *anxious, reserve, nervous* and *little*, as well as relatively descriptive words such as *hair* and *brown*. Male faces, however, had loadings for words such as *angry, moody, criminal, annoyed, scary, unfriendly, grumpy* and *unapproachable*. While the topics within sexes show the same kind of positive and negative valence impressions, male faces receive a greater variety of negative descriptions. Visualizations of the facial characteristics associated with the topics for male and female faces are shown in Figure 1c.

#### Sentiment analysis of male and female topic structures

The topic structures for female and male faces are broadly similar but show some qualitative differences in the loading of words used. For example, for Topic 2, a wider variety of negatively valanced words appear to be used to describe male faces. To investigate these differences further, we employed a sentiment analysis of the top 20 highest loading words for each topic, separately for female and male faces. Sentiment analysis uses dictionary‐based methods (Hutto & Gilbert, 2015) to score the valence of written text from −1 (extremely negative) to 1 (extremely positive). We submitted the top 20 words of each topic, for each sex, to sentiment analysis and then tested for differences in average sentiment between conditions using a Bayesian linear regression model. Specifically, the model tested the interaction between sex and topic. We coded the sex and topic variables to have the reference values ‘female’ and ‘Topic 1’, respectively, and as such, the interaction coefficient indicates the *difference‐in‐difference* (i.e., whether the average difference in sentiment scores between males and females is greater for males under Topic 2).

The model indicated a clear effect of topic, such that the words loading highly on Topic 2 had a lower sentiment score on average than those in Topic 1, with the 94% credible area of the posterior distribution of the coefficient comfortably excluding zero, *b* = −0.38 [−0.53, −0.22]. That is, Topic 2 sentiment was approximately −0.38 units lower than Topic 1, on average. In addition, the model indicated that the words for the male topic model very likely received lower sentiment on average than the female topic model, *b* = −0.15 [−0.31, 0.01], with a probability of 97% for a negative effect. Male faces received on average − 0.15 sentiment units less than female faces, regardless of topic. However, the interaction term showed no clear evidence of males having credibly different sentiment scores for Topic 2 as compared to females, *b* = −0.03 [−0.24, 0.20], with a probability of just 41% of being positive. These results are shown in Figure 1d,e and indicate that the difference in means between Topic 1 and Topic 2 is credibly similar in magnitude for both female and male faces.

#### Interim discussion

Using natural language processing techniques, we uncovered the emergence of a two‐topic structure from written first impressions of a large sample of face images. These topics were generated from spontaneously written first impressions and seem to broadly capture aspects of positive emotional valence, warmth and approachability (Topic 1) and negative emotional valence and potential threat (Topic 2). The two topics were negatively correlated. Splitting the dataset by face sex revealed that a similar two‐topic structure emerges for both female and male faces, with some substantial overlap in the key words used for each topic.

## STUDY 2 – COMPARISONS OF TEXT AND RATING‐BASED MODELS OF FACIAL FIRST IMPRESSIONS

Analysis of free‐text descriptions of faces in Study 1 suggested that written descriptions of first impressions are underpinned by two topics that appear to primarily reflect impressions of positive emotional valence, warmth and approachability (Topic 1) and impressions of negative emotional valence and potential threat (Topic 2). As these dimensions are conceptually similar to the Valence and Dominance components previously found to underpin explicit ratings of faces (Jones & Kramer, 2021; McAleer et al., 2014; Morrison et al., 2017; Oosterhof & Todorov, 2008), Study 2 investigated the relationships between the topics derived from free‐text descriptions in Study 1 and components derived from PCA of explicit trait ratings of the same face images. Specifically, we instantiated the Valence–Dominance model for these faces by conducting a PCA on ratings of the same traits used in previous studies that generated the valence–dominance model of first impressions (Jones et al., 2021; Oosterhof & Todorov, 2008).

### Method

Ratings of the 2222 faces used in Study 1 for the traits *attractive, unhappy, sociable, emotionally stable, mean, boring, aggressive, weird, intelligent, confident, caring, egotistic, responsible* and *trustworthy* (14 of the 15 traits used to derive the valence–dominance model; Oosterhof & Todorov, 2008) were publicly available from their original study (Bainbridge et al., 2013). Because the traits used to derive the valence–dominance model in previous work also included the additional trait of dominance, we recruited 225 additional participants (mean age = 38.94 years, *SD* = 14.27 years, 132 females) who each rated one of 22 subsets of images (101 images per image subset) for dominance using a 1 (not very) to 7 (very) scale (average number of raters per face = 10.23, *SD* = 0.42), with trial order fully randomized. These additional participants (i.e., those who rated the images for dominance) were recruited via Prolific with the same constraints on recruitment as Study 1. Intraclass correlations for these traits varied from 0.26 to 0.44 (see Figure S1).

Following previous studies of the Valence–Dominance model (Jones et al., 2021; Oosterhof & Todorov, 2008), we carried out a PCA on the averaged ratings for each trait per face, retaining the first two components only. We then regressed these components onto the loadings each face received for the two topics obtained in Study One, using Bayesian linear regression and adjusting for the sex of each face.

### Results

#### Valence dominance model extraction

The full correlation structure of the PCA is shown in Table 1. The first principal component explained 59% of the variance in trait ratings and showed high correlations with traits such as *unhappy* (*r* = 0.91), *caring* (*r =* −0.91), *sociable* (*r* = −0.89) and *trustworthy* (*r* = −0.90), while the second component explained 12% of the variance and was highly correlated with *dominance* (*r* = 0.69) and *egotistic* (*r* = 0.61). This pattern of results aligns closely with those reported in previous studies of the valence–dominance model (64% and 18% variance explained in the first and second components, respectively, Oosterhof & Todorov, 2008). As such, we refer to the first component as Valence and the second as Dominance. Examination of the rest of the principal components showed that the third principal component explained 8.1% of the variance (like the 6% explained by the third component found in the initial valence–dominance model extraction; Oosterhof & Todorov, 2008). Beyond this, each component explained less than 4.2% of the successive variance. Taken together, the first and second components captured 71% of the variance in the trait ratings data.

**TABLE 1:** Loadings of individual traits with each component of the valence–dominance model.

Trait	Valence–dominance model	
	PC1	PC2
Unhappy	**0.91**	0.02
Caring	**−0.91**	−0.22
Sociable	**−0.89**	0
Attractive	**−0.6**	0.36
Confident	**−0.69**	0.57
Dominance	0.02	**0.69**
Mean	**0.90**	0.25
Intelligent	**−0.74**	0.27
Weird	**0.76**	−0.24
Trustworthy	**−0.9**	−0.12
Egotistic	**0.61**	**0.61**
Boring	**0.56**	−0.34
Responsible	**−0.84**	0.05
Aggressive	**0.84**	0.36
Emotionally Stable	**−0.86**	0.02

*Note*: Values above |.5| are highlighted in bold.

Our PCA was calculated using a singular value decomposition of the correlation matrix between the 15 trait ratings and then ordering the singular values in decreasing order. No rotations were carried out (i.e., principal components retain their maximal‐variance properties).

#### Mapping the valence dominance model to the topic model

Since the direction of principal components is arbitrary, we rescaled the first PC (Valence) so that higher values represented more positive valence (i.e., altering only the sign of the correlations stated above). We *z*‐score standardized all variables beforehand, except for face sex (coded one for male), and fit a model that predicted topic loadings with no intercept, the main effect of each principal component (PC1 = Valence, PC2 = Dominance), the main effect of each topic (whether a loading belonged to Topic 1 or Topic 2), and the interactions between each principal component and each topic. Face sex was included as a covariate.

By standardizing all variables and removing the intercept, we can directly interpret the coefficients as standard deviation unit changes in topic loadings with a 1SD increase in loadings on the principal component. We used weakly regularizing normal priors on each coefficient (mean zero and standard deviation one, Gelman et al., 2020) and a *t*‐distributed likelihood to be robust to the influence of outliers (Kruschke, 2014).

**FIGURE 2:**
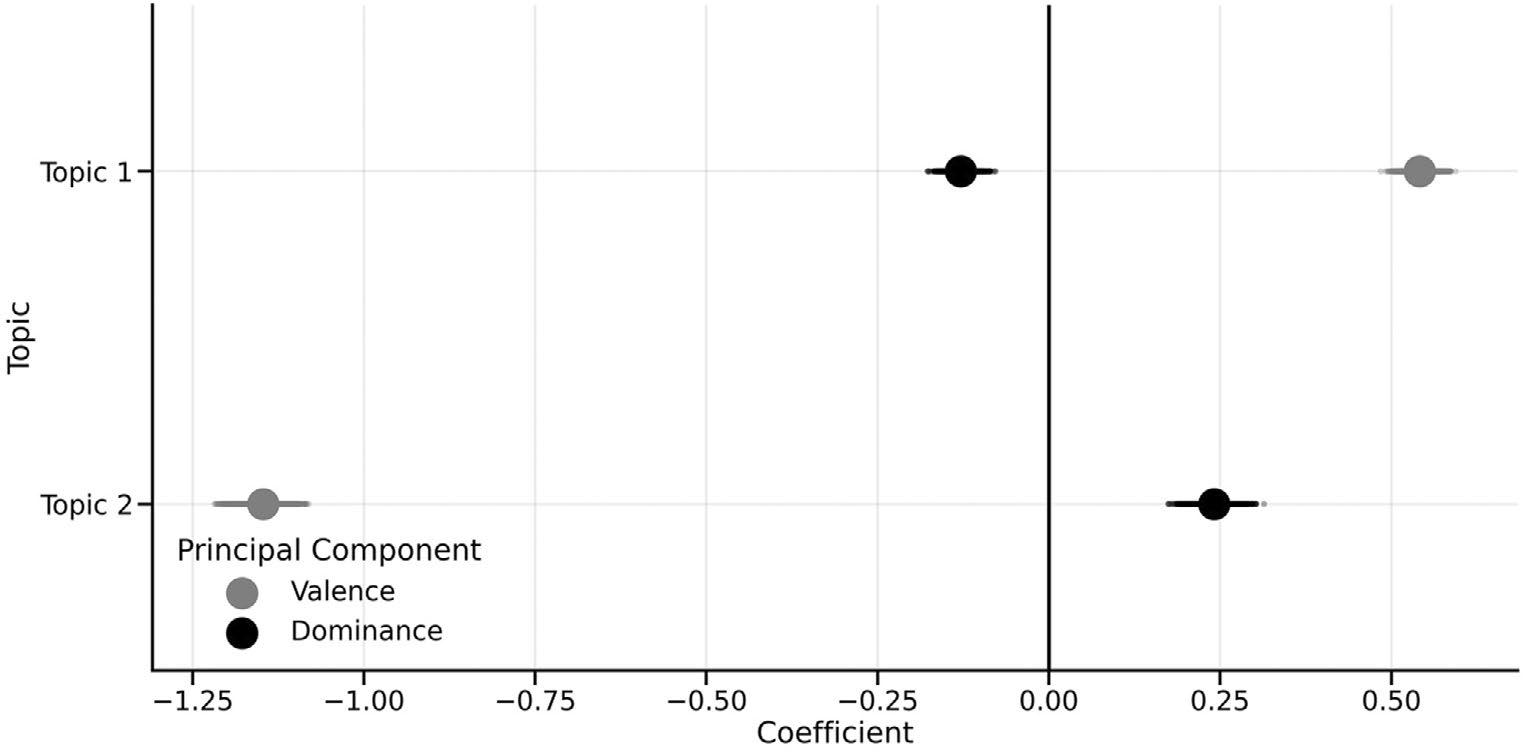
Coefficients and 94% credible intervals for the association between each principal component and the loadings on each topic.

Adjusted for sex, the valence–dominance model explained 44.4% [43.2, 45.7] of the variance in topic loadings. Examining the coefficients showed clear and strong associations between each component and each topic loading. For Topic 1, increases in PC1 (Valence) were associated with positive loadings, *b* = 0.54 [0.52, 0.57], and increases in PC2 (Dominance) were associated with lower loadings, *b* = −0.13 [−0.16, −0.10]. For Topic 2, increases in PC1 were associated with much lower loadings, *b* = −1.15 [−1.19, −1.11], while PC2 showed a smaller but positive relationship, *b* = 0.24 [0.21, 0.28]. The posterior distributions of each coefficient comfortably excluded zero and ranged in size from approximately one tenth of an SD change in topic loadings (PC2's association with Topic 1) through to just over one SD unit (PC1's association with Topic 2). These results indicate clear associations between the valence–dominance model and the topic loading model. However, under half of the variance in the topic loadings was accounted for by the valence–dominance model. Coefficients are shown in Figure 2.

## GENERAL DISCUSSION

In Study 1, topic modelling of free‐text descriptions of participants' first impressions of 2222 face images revealed that these impressions were underpinned by two topics (i.e., dimensions), reflecting perceptions of positive emotional valence, warmth and approachability (Topic 1) and perceptions of negative emotional valence and potential threat (Topic 2). This pattern of results suggests that free‐text descriptions of first impressions contain consistent statistical regularities that can be extracted using natural language processing (NLP) methods. Importantly, the same pattern of results was observed when free‐text descriptions of all images were analysed and when descriptions of male and female images were analysed separately, suggesting that the pattern of results is not simply a by‐product of differences in how participants described male and female faces.

Studies that derive models of first impressions by analysing explicit trait ratings of face images have typically reported that first impressions are underpinned by Valence and Dominance components that are either orthogonal or weakly correlated (e.g., Jones et al., 2021; Oosterhof & Todorov, 2008). In Study 2, we replicated this pattern of results in our analyses of explicit trait ratings. However, our analyses in Study 2 also suggested that there is a potentially important quantitative distinction between models derived from these two types of data. Collectively, the Valence and Dominance components derived from analyses of explicit trait ratings of face images explained ~44% of the variance in the topics extracted from free‐text descriptions. This suggests that, although there is some overlap in the dimensions underpinning first impressions derived from these two types of responses, models derived from explicit ratings do not fully capture the language that participants typically use to describe first impressions. Existing models of first impressions are non‐redundant with topic models, and natural language‐based approaches highlight many aspects of first impressions that are unrelated to personality, as indicated by the range of words related to emotional states and physical appearance. As such, natural language offers an alternative approach to studying first impressions and offers support to the current literature, as well as novel insights into the complexity of the process. Moreover, it offers a ‘high fidelity’ method of studying first impressions and similar approaches, being able to capture rich information about processes as they are spontaneously produced, as opposed to forcing observers to make judgements of specific traits.

That the Valence and Dominance components derived from analyses of explicit trait ratings of face images explained ~44% of the variance in the topics extracted from free‐text descriptions may have relatively straightforward explanations. First, the traits used to estimate the Valence–Dominance model based on Oosterhof and Todorov's (2008) approach were based on standardized images. As naturally varying images were used here, these traits may not capture the impressions attributed to these more variable images. However, we closely reproduce the Valence–Dominance model in this data, and it is unlikely the model would appear only under standardized conditions. An alternative explanation is that the traits used to generate the Valence–Dominance are already filtered down to just personality‐related first impressions, and not first impressions in general. For example, some words captured by the topic model include physical descriptors (*old, smiley*) and emotional states (*angry, sad*), which go beyond the content of the Valence–Dominance model, and as such, that model may be unable to capture information about general impressions present in text data. While the data‐driven approach could in principle be expanded to include non‐personality‐related words by collecting ratings, it would require an increase in the number of traits to be rated by additional participants. Working directly with text data captures aspects of first impression that are ignored in traditional approaches.

However, the difference can also be explained by a fundamental distinction between ‘can’ and ‘do’ approaches in psychology (Satchell et al., 2022). Explicit trait ratings are clearly useful approximations of first impressions, but a significant limitation is that they ‘coerce’ participants into considering a judgement of a trait that they may not have spontaneously generated. To elaborate, observers can make ratings of traits like *emotional stability* when explicitly prompted to rate stimuli on that trait, but such trait ratings may not map straightforwardly to the topics generated from free text, which constitute the impressions that observers form in the moment they view the face. Thus, text‐based approaches may offer a new route to advance models of social perception (Mondloch et al., 2023), as they can capture actually‐formed impressions moments after that impression is formed. By contrast with previous research that has used text‐based methods to generate a list of traits for further observers to rate (Oosterhof & Todorov, 2008; Sutherland et al., 2018), more directly analysing text responses themselves also avoids the assumption that a different set of participants would necessarily spontaneously generate similar responses. By using natural language, a complex process such as first impressions can be modelled more fully, and in doing so we find that various features that are unrelated to personality judgements are relatively common – social impressions encompass more than just personality judgements.

The extracted topic models show broad theoretical similarities to existing models of social perception. For example, the words people use to describe their impressions of others can be distilled into a smaller number of features (i.e., topics), a feature of many social cognitive models outside of face perception. The stereotype content model – underpinned by the dimensions of warmth and competence or the ‘Big Two’ axes of social cognition (Fiske, 2018; Fiske et al., 2007) – emerges across varied domains of psychological science and reflects core aspects of functioning in a social world (Martin & Slepian, 2021). More specifically, these two dimensions often appear as variations around concepts such as agency and communality (Eagly, 1997), particularly behaviours focused on self‐interest, promotion and preservation, or community and prosociality, respectively (Ybarra et al., 2008). The content of Topic 1 aligns closely with the latter dimension, with language describing smiling, positive and prosocial appearances. The content of Topic 2 aligns particularly with agentic concepts and the identification of individuals who pose threats or harm or are displaying negative emotions. The topics also lend support to the recent idea that the fundamental axes of social cognition reflect a functional, gendered perception of the social world (Martin & Slepian, 2021). Though a two‐topic solution emerged for both female and male faces when considered separately, there were divergences and overlaps between the words used for each of those topics. For example, in Topic 2, the words *anxious* and *nervous* were exclusively used for female faces, while male faces were described using words such as *angry* and *scary*, which suggests the kinds of positive and negative valence perceived are adjusted to some extent conditionally on the sex of the observed individual.

The results also point to an update in our understanding of models of facial first impressions. Traditional data‐driven models based on ratings posit aspects of valence and dominance as separate components that may be orthogonal (Oosterhof & Todorov, 2008; Sutherland et al., 2013) or cast other impressions such as competence and warmth into separate components (Lin et al., 2021). With the high‐fidelity nature of text data, the derived topic model here suggests that positive and negative valence emerge as separate dimensions on their own but are strongly correlated. This pattern differs from existing models in that it suggests valence is a primary ‘thread’ that ties first impressions together in a low‐dimensional space. These results align with a general and fundamental ‘approach or avoid’ continuum that underpins perception, governing whether a stimulus or conspecific should be moved towards or away from (Allport, 1935; Bamford & Ward, 2008; Chen & Bargh, 1999; Jones & Kramer, 2021). Similar findings have emerged using clustering methods that, based on trait ratings, partition faces into a fundamental ‘approach or avoid’ decision (Jones & Kramer, 2021). The topic model here may suggest that what underpins first impressions is an evaluation that leads to a binary decision about whether to approach or avoid a conspecific.

In the current work, we deliberately did not constrain free‐text responses in any way. A limitation of this approach was that, without instruction, participants tended to produce relatively short free‐text descriptions of individuals. Thus, while our analyses suggest that models derived from unconstrained free‐text descriptions and explicit trait ratings show conceptual – but nonredundant – overlap, it remains an open question whether more constrained free‐text descriptions would produce topics identical to those seen here. Systematically varying the amount of text participants are required to write in each description would be needed to clarify this issue. Varying the context in which people are asked to generate free‐text descriptions (e.g., instructing participants that the individuals shown are job applicants versus potential romantic partners) would also allow the generalizability of the topics across assessment contexts to be probed.

To conclude, we show for the first time that the statistical regularities in free‐text responses of facial first impressions are underpinned by two topics that reflect perceptions of positive emotional valence, warmth and approachability (Topic 1) and negative emotional valence and potential threat (Topic 2). Components derived from explicit trait ratings of face images explain ~44% of the variance in the topics extracted from free‐text descriptions, demonstrating that models built from free‐text responses are non‐redundant with existing models of first impressions derived that rely on ratings and demonstrating the utility of natural language as a vehicle for more closely studying first impressions. The derived topic model here suggests a subtly different psychological organization of facial first impressions, suggesting separate components for both positive and negative valence, which subsume separate or orthogonal components from existing approaches. Future work can compare how natural language‐based models and traditional data‐driven approaches can explain impressions in varying social contexts or whether the blending of these models can offer a greater understanding of first impressions. More generally, a key avenue of investigation is how natural language processing can advance and refine models of social cognition.

## AUTHOR CONTRIBUTIONS

**Alex L. Jones:** Conceptualization; investigation; writing – original draft; methodology; visualization; writing – review and editing; software; formal analysis; data curation. **Victor Shiramizu:** Investigation; writing – review and editing; resources; data curation. **Benedict C. Jones:** Investigation; funding acquisition; writing – original draft; writing – review and editing; validation; project administration.

## CONFLICT OF INTEREST STATEMENT

All authors have no conflicts of interest to declare.

## Supporting information


**Data S1.** Supporting Information.

## Data Availability

The data that support the findings of this study are openly available in OSF at https://osf.io/chxnp/.
