# Supplementary material for: Decoding the language of first impressions: Comparing models of first impressions of faces derived from free‐text descriptions and trait ratings
Source: Br J Psychol. 2024 Jun 17;117(2):725–40. doi: 10.1111/bjop.12717 (PMC13051011; doi:10.1111/bjop.12717)
Supplement: Supplementary file 1 — Data S1. Supporting Information. [file BJOP-117-725-s001.docx]

**Supplementary materials**

**Study One Supplemental Analyses**

**Deriving Topics with Latent Semantic Analysis**

Our main topic model was estimated using non-negative matrix factorisation (NMF) which indicated a two-topic structure as an optimal solution, capturing a positive and negative valence dimension. NMF has the advantage of retrieving simple, ‘parts based’ representations of complex data, and has interpretable non-negative loadings (i.e., faces or words can be associated positively with a component but at minimum have a zero loading). However, this property may impact the structure of the resulting topics in an arbitrary way that differs from how trait rating based approaches function. For example, consider that a principal components analysis of the trait ratings of ‘*trustworthy*’ and *‘untrustworthy’* would load these variables onto a single component, one high and one low, capturing valence as opposite ends of a single continuum. Conversely, as NMF disallows negative loadings, it may need more components to capture what could psychologically be a single dimension, as PCA-based models suggest.

To address this potential artefact, we employed another technique in natural language processing, known as Latent Semantic Analysis (LSA), which is a singular-value decomposition (SVD) of a TF-IDF representation of text. SVD - which is also the basis of PCA – produces word loadings on components (i.e., topics) which can vary from positive to negative, and additionally forces those components to be orthogonal. Like NMF it produces both a word-topic loading matrix and a document-topic loading matrix, but additionally generates the singular values array that describes the relative ‘strength’ of the topics. This approach ideally allows us to test the generalisability of the topic model structure and whether the positive and negative valence components appear under a different set of mathematical constraints.

**Identifying number of topics with LSA.** We repeated the same analytical pipeline as in the main analysis, carrying out LSA with a range of possible topics from two to six, and evaluating the coherence of the words with the 20 highest absolute loadings on the topic. We find that two topics again produced the most coherent fit (two topics UMass = -2.50, three topics UMass = -3.02, four topics UMass = -2.74, five topics UMass = -2.83, six topics UMass = -3.19).

**Examining a two-topic LSA model.** Extracting a two-topic structure revealed that Topic 1 had only positive loadings (minimum = 0.00, maximum = 0.71), and contained positively valanced words, almost identical to the first topic of the NMF model. The 20 words with the highest absolute loadings were: *happy, friendly, kind, smile, confident, nice, warm, fun, shy, cheerful, approachable, funny, good, content, old, eye, professional, serious, outgoing,* and *open*. This closely replicates the first topic of the main analysis.

Topic 2 had both positive and negative loadings (minimum = -0.32, maximum = 0.71), and the 20 words with the highest absolute loadings were almost entirely negatively valanced, being: *serious, sad, happy, angry, unhappy, shy, worried, unsure, moody, thoughtful, neutral, confident, eye, quiet, annoyed, criminal, tired, scary, intense,* and *stern*. This is very similar to the second topic of the main analysis, capturing aspects of negative emotion and threat potential. Given that this topic had positive and negative loadings, we examined the top and bottom 10 words to try best characterise this topic dimension. The top 10 words, as expected, were generally negatively valanced: *serious, sad, angry, unhappy, shy, worried, unsure, moody, thoughtful,* and *neutral.* Interestingly, the bottom 10 words captured positive valence, suggesting LSA characterises this component as a unidimensional valence dimension: *happy, friendly, fun, cheerful, joyful, bubbly, outgoing, jolly, warm,* and *smiley*, albeit with a significantly stronger emphasis on negative valence – only *happy* had a large enough absolute loading value to make it to the top 20 words for this topic.

However, examination of the singular values showed that Topic 1 had almost twice the relative strength of Topic 2 (10.81 and 5.71, respectively), which indicates a primacy of positive valence descriptions in first impressions, followed by negative valence.

As such, a different mathematical approach to generating topic models confirms our initial result that a two-topic structure underpins the natural language of first impressions. To quantify this exactly, we obtained the loadings each face received on both topics under both topic modelling methods (NMF and LSA), and correlated them. Topic 1 loadings were almost perfectly correlated between models, *r* = .99 [0.984, .996], while Topic 2 loadings were only slightly less so, *r* = .96 [0.947, .970], indicating extremely high agreement between topic models.

**Cross-Validation of the Two-Topic Structure**

To test the robustness of the two-topic structure that emerged in the full dataset, we carried out split-half cross validations. As topic modelling is an unsupervised learning task, cross validation is not as straightforward as with supervised approaches.

Our strategy involved splitting the corpus of 2,222 text descriptions (merged across participants, as in the main study) in half, and preprocessing the text in the same way as in the main study to create a TF-IDF array, separately in each half. Each of these arrays were then decomposed with a two-component NMF model as in the main study, and we extracted the top 20 highest loading words for each component. We then used the Ochiai coefficient, a set-based measure of cosine similarity that varies between zero (completely different) and one (perfectly similar) to assess the degree of similarity between the top 20 words for each topic in each split. The Ochiai coefficient measures the number of tokens (i.e., words) present in both A and B, divided by the square root of the product of the number tokens in A and B separately:

$$S= \frac{\left| A\cap B \right|}{\sqrt{\left| A \right|\left| B \right|}}$$

Using this coefficient, we compared the highest loading words in Topic 1 in the first half split to the highest loading words in Topic 1 in the second half split, and so on. We also applied this coefficient to the top 20 words for each topic, under each split, to the top 20 words that emerged from the topic model in the full dataset.

We repeated this entire procedure (splitting, preprocessing, creating TF-IDF arrays, NMF decomposition, and similarity assessment) 1,000 times, and compute the average and 94% highest density intervals of the resulting similarity coefficients. This cross-validation allows us to assess two things – first, whether similar topic content emerges with repeated random splits of the data, and second, whether repeated random splits of a dataset half the size of the original results in a topic structure akin to the original.

The results of the between-split topic similarities (i.e., comparing one half to the other for each topic) are shown in Figure S1. Topic 1 showed consistently high similarity between halves (mean = 0.85 [0.80, 0.95]), while Topic 2 showed good but lower levels of similarity (mean = 0.66 [0.55, 0.75]).

When assessing the similarity for each topics top 20 words within each split to those observed in the full data, we observed high agreement. Topic 1 showed very high similarity (first half association with full model; mean = 0.92 [0.85, 1], second half; mean = 0.92 [0.85, 1]), as did Topic 2 with a lower similarity (first half association with full model; mean = 0.83 [0.75, 0.95], second half; mean = 0.83 [0.75, 0.95]).

Our results suggest that the topics we observed in the main analysis exhibit stability, particularly for Topic 1. While Topic 2 has somewhat lower stability, it still exhibits similarity in line with the full dataset results.


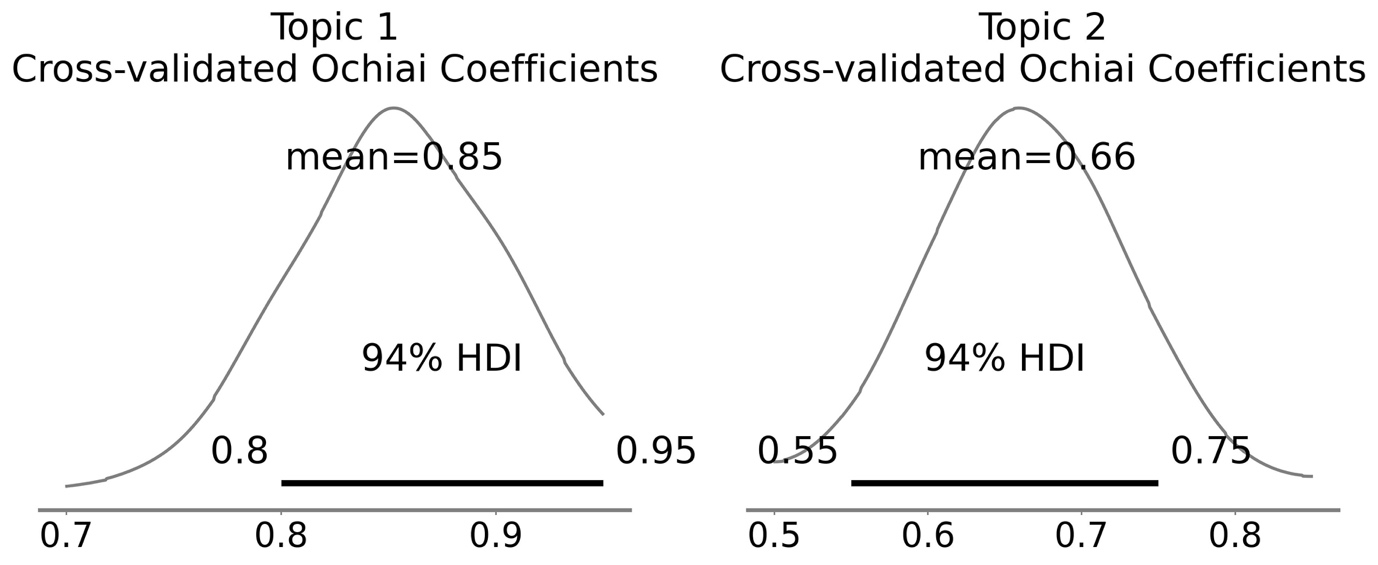


**Figure S1.** Distributions of split-half cross validation Ochiai coefficients, assessing the similarity of top-loading topic words between each split-half.

**Study Two Supplemental Analyses**

**Intraclass Correlations for Trait Ratings**

To estimate the ICC for each trait, we took the raw, disaggregated data for each explicit trait rating, and fitted an intercept only model with random effects for both faces and participants, using Bayesian estimate. In the Bayesian framework this equated to taking the standard deviations of the hyperpriors for the face and participant effects, adding them, and dividing them by the sum of both as well as the standard deviation of the model’s error variance (McGraw & Wong, 1996). The results are shown in Figure S1 and Table S1.

**
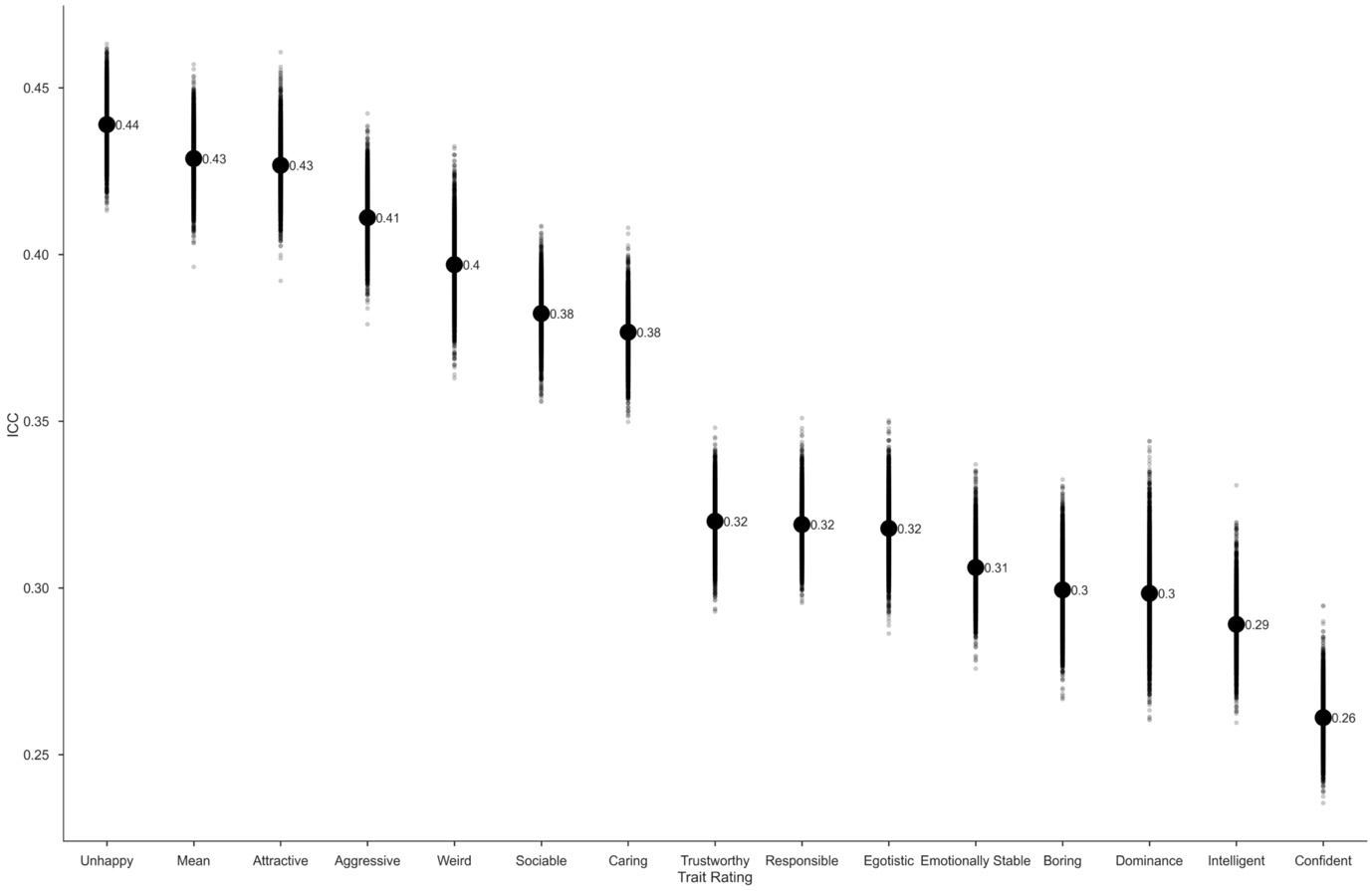
**

**Figure S1.** Intraclass correlations estimated from the posterior distributions of intercept-only Bayesian mixed effects models.

**Table S1. Intraclass Correlation Posterior Distributions.**

| Trait | **ICC (mean)** | Lower Bound 94% CrI | Upper Bound 94% CrI |
| --- | --- | --- | --- |
| **Aggressive** | 0.41 | 0.40 | 0.43 |
| **Attractive** | 0.43 | 0.41 | 0.44 |
| **Boring** | 0.30 | 0.28 | 0.32 |
| **Caring** | 0.38 | 0.36 | 0.39 |
| **Confident** | 0.26 | 0.25 | 0.28 |
| **Dominance** | 0.30 | 0.27 | 0.32 |
| **Egotistic** | 0.32 | 0.30 | 0.33 |
| **Emotionally Stable** | 0.31 | 0.29 | 0.32 |
| **Intelligent** | 0.29 | 0.27 | 0.31 |
| **Mean** | 0.43 | 0.41 | 0.44 |
| **Responsible** | 0.32 | 0.31 | 0.33 |
| **Sociable** | 0.38 | 0.37 | 0.40 |
| **Trustworthy** | 0.32 | 0.31 | 0.34 |
| **Unhappy** | 0.44 | 0.42 | 0.45 |
| **Weird** | 0.40 | 0.38 | 0.41 |

**Mapping the Valence Dominance Model to the LSA Topic Model**

While we observed the same two-topic structure when employing LSA, there were some subtle differences, such as Topic 2 showing negative and positive loadings, and appearing as a kind of unidimensional valence dimension that was in addition to the strong positive valence of Topic 1. Despite this, the loadings faces received on the topics, under each topic model structure, were extremely correlated. Nonetheless, to see whether these small differences impact our overall conclusions, we replicated the analysis of Study Two, using the principal components derived from trait ratings to this time predict the loadings on the LSA derived topics instead. The model specification was identical to that of the main analysis, and given the tightly correlated nature of the topic loadings, the results were large similar.

For the LSA-derived Topic 1, increases in PC1 (Valence) were associated with positive loadings, *b* = 0.53 [0.50, 0.56], and increases in PC2 (Dominance) were associated with lower loadings, *b* = -0.13 [-0.15, -0.10]. For LSA-derived Topic 2, increases in PC1 were associated with lower loadings, *b* = -1.17 [-1.21, -1.13], but PC2 showed a smaller but positive relationship, *b* = 0.25 [0.21, 0.29]. This pattern of coefficients is virtually identical, and the principal components consequently explained an almost identical proportion of variance in these topics as in the NMF derived topics, 44.6% [43.4, 45.8].
